# Spontaneous Human Combustion

**Published:** 1848

**Authors:** 


### ARTICLE V.

### *Spontaneous Human Combustion.*

The "Gazette Médicale" quotes from the "Union Médicale" the following case of alleged spontaneous combustion. On the morning of the 6th of January, 1847, the body of a man named Ch—— was found on fire in bed. A dense smoke filled the room. One who was present affirmed that he saw on the body of the deceased, a small, lambent, whitish flame. All the bedclothes and clothes of the deceased were almost entirely destroyed. The bedstead was only partly burnt; there were no ashes, and very little vegetable charcoal, but some portions of animal charcoal having evidently belonged to the articulations. The other materials surrounding the body were scorched. It is said that M. C—— carried in his waistcoat pocket some chemical matches, and in the evening he had, as usual, placed at his feet a heated brick, which, before being wrapped in linen, had been slowly cooled by water thrown over it twice. He went to his room between six and seven o'clock in the evening. Two hours later, his son and daughter-in-law, passing his door, perceived nothing unusual; and it was not till the next morning, that his grandson found him in the state which we have described. He was 71 years of age, and was neither very fat, nor given to drunkenness. The weather had been very cold for some time, but there were no signs of an excess of atmospheric electricity. The body was found in its usual position during sleep. His son and daughter were suspected of having first murdered him, and then burnt the body, in order to conceal all traces of the crime. Dr. Masson, who was ordered by the authorities to make the necessary examination, had the body exhumed. The coffin was found half filled. The body was folded in a white shroud. A cravat, nearly destroyed by the fire, and a fragment of a shirt collar, remained round the neck. The hands, burnt to a cinder, were attached to the forearm merely by some carbonized tendons, which gave way at the least touch. Lastly, the thighs were so completely separated, that, had it not been for fragments of animal charcoal, the separation might have been attributed to a knife.

From the examination of these facts, it was concluded that, as it was impossible to attribute the phenomena to the action of the combustibles with which the body had been in contact, they must be ascribed to a cause inherent in the individual, put in action, perhaps, by the heat of the brick applied to the feet, but which must have found fuel in the tissnes which it destroyed; that, in a word, it must be classed among cases of spontaneous combustion. This opinion of M. Easson being fully confirmed by that of M. Orfila, the accused were acquitted.—*Ranking's Abs.*

